# Postnatal screening and care for non-medical risk factors by preventive child healthcare in deprived and non-deprived neighbourhoods

**DOI:** 10.1186/s12913-018-3243-2

**Published:** 2018-06-08

**Authors:** M. R. C. van Minde, S. M. Hulst, H. Raat, E. A. P. Steegers, M. L. A. de Kroon

**Affiliations:** 1 Department of Obstetrics & Gynaecology, Division of Obstetrics and Prenatal Medicine, Erasmus University Medical Centre, Rotterdam, The Netherlands; 2 Department of Public Health, Erasmus University Medical Centre, Rotterdam, The Netherlands; 3 Department of Health Sciences, University Medical Centre Groningen, Groningen, The Netherlands

**Keywords:** Preventive child healthcare, Non-medical risk factors, Risk screening practices, Postnatal risk assessment, Vulnerable families, Deprived neighbourhoods

## Abstract

**Background:**

Children born in families with non-medical risk factors, such as deprivation, have higher odds of preterm birth (< 37 weeks of gestation) or being born small for gestational age (birth weight < 10th percentile). In addition, growing up they are at risk for growth and developmental problems. Preventive Child Healthcare (PCHC) monitors growth and development of babies and children. Early identification of children at risk could result in early interventions to prevent growth and developmental problems in later life. Therefore, we aimed to assess current practices in postnatal risk screening and care for non-medical risk factors and the collaboration with other healthcare professionals, in both deprived and non-deprived neighbourhoods in the Netherlands.

**Methods:**

Eight out of ten invited PCHC organisations, from different areas in the Netherlands, consented to participate in this study. A questionnaire was designed and digitally distributed to professionals working at these organisations, where 370 physicians and nurses were employed. Data was collected between June and September 2016. Descriptive statistics, chi square tests and *t*-tests were applied.

**Results:**

Eighty-nine questionnaires were eligible for analyses. Twenty percent of the respondents were working in a deprived neighbourhood and 70.8% of the respondents were employed as nurse. Most of them performed screening for non-medical risk factors in at least 50% of their consultations. PCHC professionals working in deprived neighbourhoods encountered significantly more often families with non-medical risk factors and experienced significantly more communication problems than their colleagues working in non-deprived neighbourhoods. 48.2% of the respondents were satisfied with the current form of postnatal risk screening in their organisation, whereas 41.2% felt a need for a structured postnatal risk assessment. Intensified collaboration is preferred with district-teams, general practitioners and midwifes, concerning clients with non-medical risk factors.

**Conclusion:**

This study shows that postnatal screening for non-medical risk factors is part of current PCHC practice, regardless the neighbourhood status they are deployed. PCHC professionals consider screening for non-medical risk factors as their responsibility. Consequently, they felt a need for a structured postnatal risk assessment and for an intensified collaboration with other healthcare professionals.

**Electronic supplementary material:**

The online version of this article (10.1186/s12913-018-3243-2) contains supplementary material, which is available to authorized users.

## Background

The developmental theory of health and disease identified the first 1000 days (from conception to the age of 2 years) as a critical and sensitive period for the development of a human being [1]. Initial vulnerability for future disease can be aggravated by growing up in an unfavourable socio-economic environment or by other non-medical risk factors, such as lack of social support or domestic violence, affecting a child’s growth and development [1, 2]. Parental lifestyle factors such as smoking, substance abuse (drugs and alcohol) and obesity are also considered as non-medical risk factors [3] and individually influence growth and development of children [4–7]. Medical risk factors such as preterm birth and being born small for gestational age (SGA) are independently associated with a high risk for growth and developmental problems in children [8–10]. Additionally, in deprived neighbourhoods these medical risk factors are more common [11]. Both medical and non-medical risk factors, the accumulation and the interaction of these risks explain the difference in perinatal and child health among deprived and non-deprived neighbourhoods [12–14].

In the Netherlands, Preventive Child Healthcare (PCHC) organisations are responsible for monitoring child growth and development and of the promotion of healthy lifestyles. PCHC is offered to all children, from birth until the age of 19 years by the Dutch government, free of charge. For children in the age of zero up to 4 years old, consultations comprise of growth and developmental measurements, regular visits to the national vaccination programme and parenting advice. These consultations have high attendance rates (> 95%) [15].

To our knowledge, a structured postnatal risk assessment for growth and development, combining both medical and non-medical risk factors, does not yet exist for PCHC. However, for the early detection of developmental problems in toddlers, an instrument has been developed for the application in PCHC [16]. In obstetric care, an antenatal risk assessment has been developed and evaluated, assessing the risk of unfavourable birth outcomes in the first trimester of pregnancy [17].

Moreover, PCHC professional opinion on this subject has not been studied before. Studies on the views and needs of PCHC professionals are scarce. Häggman-Laitila et al. [18] described public health nurses views on the needs for special support of Finnish families, where the needs varied per region. Their findings correspond with the results of a qualitative study by Mundet-Tuduri et al. [19], who highlighted the different educational needs of public healthcare professionals, varying per region and organisation. Concerning the implementation of screening instruments, Garg et al. [20] highlighted the practical challenges of the use of recommended screening tools as part of developmental surveillance. They stressed on the need for further research regarding the most effective integrated models of care [20].

## Objective

We aimed to assess current postnatal risk screening and care practices for non-medical risk factors, additional to medical risk factors, in PCHC. We hypothesized that the magnitude of screening and care practices in the postnatal period, could be affected by working in a deprived or non-deprived neighbourhood. Additionally, we assessed the needs of PCHC professionals and their collaboration with other healthcare providers.

## Methods

### Study design

This study concerns a cross-sectional descriptive survey. The survey was conducted among PCHC professionals (physicians and nurses) working at eight different PCHC organisations in urban and rural regions in the Netherlands. This study is part of the Healthy Pregnancy 4 All-2 (HP4All-2) program [21]. HP4All-2 aims to enforce and facilitate continuous care for families at risk after birth by focusing on antenatal and postnatal risk assessment in combination with tailored care pathways by maternity care, PCHC and interconception care [21].

### Setting and study population

Every municipality in the Netherlands is responsible for coordinating their own PCHC services. Most municipalities organise PCHC within their Municipal Health Services, while some of them subcontract commercial healthcare organisations to carry out PCHC. Both types of PCHC organisations were included in order to reflect the current situation in the Netherlands. PCHC professionals in the Netherlands all comply with the same training conditions and they work according to the general requirements for PCHC, imposed by the Dutch government [22]. The study population consisted of PCHC nurses and PCHC physicians employed at these organisations. Recent data indicate that there are 36 different PCHC organisations in the Netherlands, providing care for children from birth until 19 years of age (often professionals work for either the age group 0–4 years old or the age group 5–19 years old) [23]. With the assistance of professionals working at organisations within the HP4All-2 network [21], we invited 10 different organisations, in both urban and rural areas across the country, to participate in our survey. We addressed healthcare professionals who work with children from zero up to 4 years old, because this age interval includes the postnatal period.

### Development of the questionnaire

**Fig. 1:**
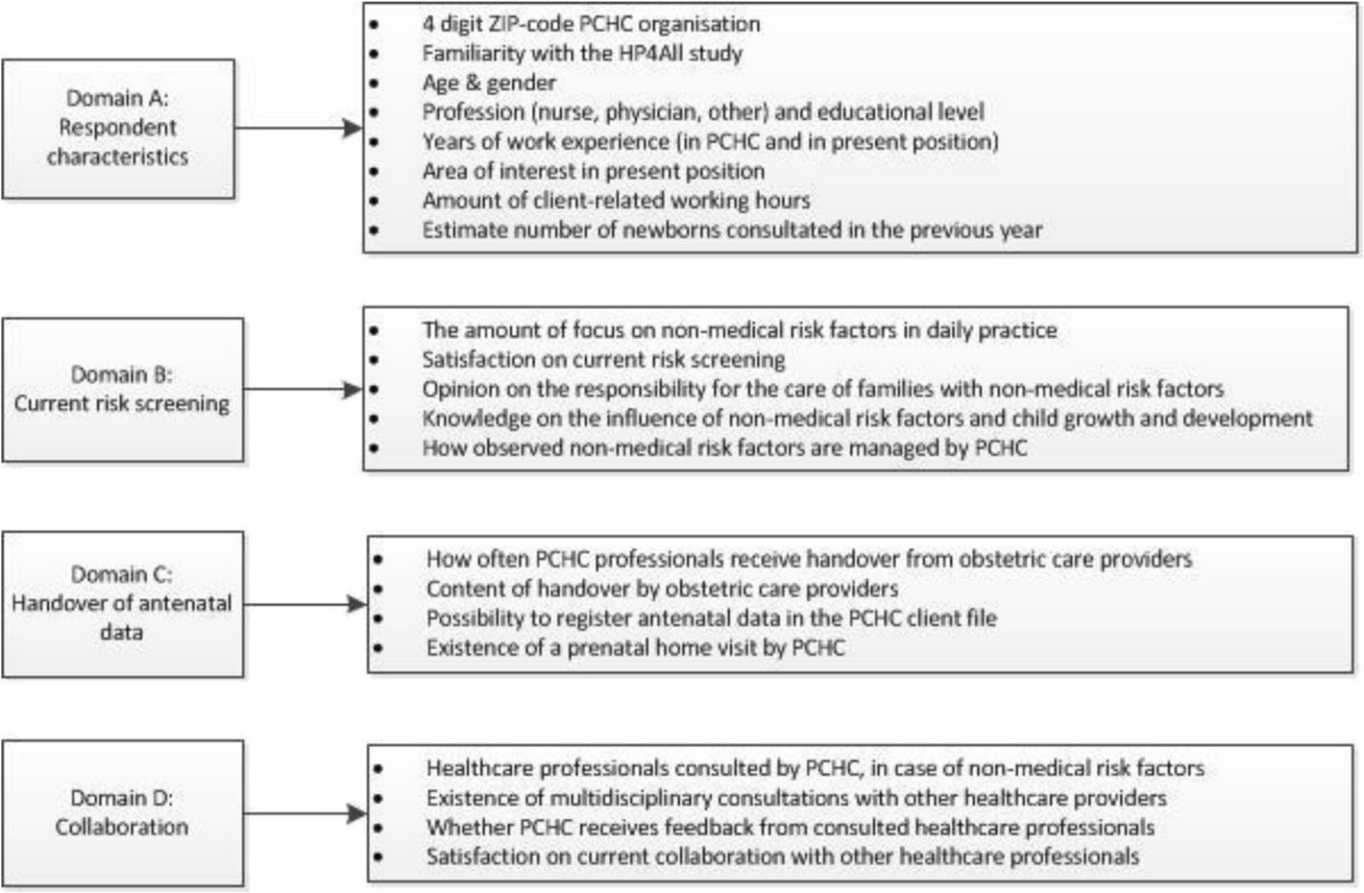
Domains, constructs and items of the questionnaire

Data were collected using an electronic questionnaire, which was developed in analogy with the validated MIDI questionnaire, an instrument to measure determinants of innovations in healthcare [24]. Finally, the questionnaire contained 41 questions, which were either closed or open-ended. The questions were divided in four domains: (A) respondent characteristics, (B) current risk screening practices (C) handover of antenatal data, and (D) collaboration with other healthcare professionals. The questions which measured the knowledge of non-medical risk factors were based on recent literature and, if available, systematic reviews or meta-analyses [4–10, 14, 25–29]. Data concerning the deprivation status of a neighbourhood in which the PCHC professional was working during the study period, was defined according to the NIVEL coding. NIVEL, the institute for health services research in the Netherlands, publishes a quadrennial overview of deprived urban areas by zip code. Every 4 years, the NIVEL institute aggregates neighbourhood-level data on the number of inhabitants, the area density by address, the proportion of non-western inhabitants, average income of residents with an income and the number of residents with social security benefits. Hence, a standardised formula is used to calculate the so-called deprivation index. Based on this deprivation index, deprived neighbourhoods are designated [30, 31]. The questionnaire was piloted among three PCHC professionals to examine whether terms and definitions were clear and precise. For its design and distribution we used the online survey program, LimeSurvey (Pro version, © 2003). A summary of the questionnaire is presented in Fig. 1. The full questionnaire can be made available upon request.

### Exclusion criteria

Preliminary exclusion criteria for analysis were not being employed as a PCHC physician or nurse, and not working with the age-group zero up to 4 years old.

### Data collection

Data were collected between May and August 2016. As soon as the PCHC organisations agreed to participate, they received an email containing the link to the electronic questionnaire in LimeSurvey. The management of the participating organisations distributed the link among their (selection of) employees and they were asked to send at least one reminder. Most PCHC organisations participated with their whole workforce, others decided to distribute the questionnaire among a selected group of employees, e.g. limited to one zip code area or neighbourhood. The managers themselves made the decision on how to distribute the questionnaire within their organisation.

### Data analyses

Descriptive statistics were applied to quantitatively describe the main features of the data. Additionally, comparative statistics were used, i.e. the chi-squared test and the Fisher’s exact test (if expected frequencies were not greater than five) to measure associations between two categorical variables. The unpaired *t*-test or Mann-Whitney U test were applied to compare ordinal or interval variables between two (in-)dependent groups. All statistical analyses were performed using SPSS software (version 20.0). Statistical significance was defined as a *p* value < 0.05.

## Results

**Fig. 2:**
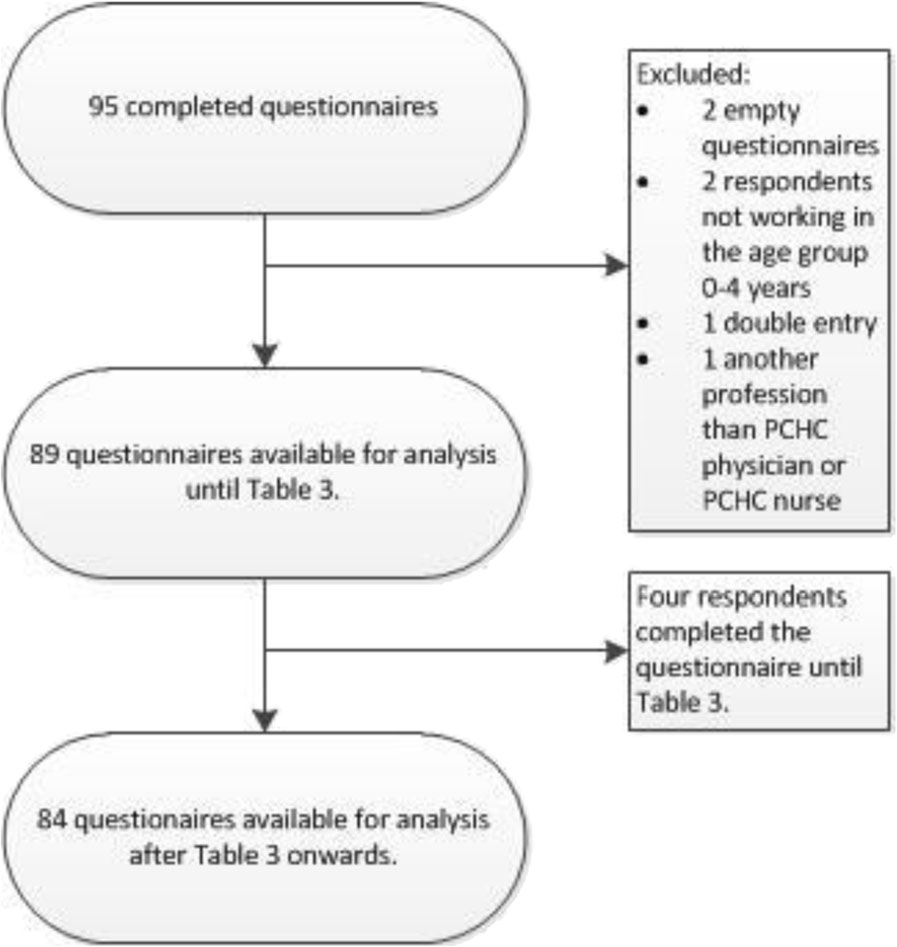
Process of inclusion and exclusion of questionnaires

Eight out of ten invited PCHC organisations agreed to participate. The eight participating organisations were nationally scattered, representing both urban and rural regions of the country. The response rate per organisation varied from 100%, being the highest, to 15,6%, being the lowest response rate; 100% reflecting an organisation which had chosen to distribute to questionnaire to the employees of one specific neighbourhood and 15,6% reflecting an organisation which had sent the questionnaire to their whole workforce. Figure 2 represents the flowchart of excluded questionnaires. Eighty-nine questionnaires remained available for analyses.

Table 1 shows the respondents’ characteristics. Twenty percent of the respondents were working in a deprived neighbourhood according to the NIVEL coding [30]. 70.8% of the respondents were nurses and 29.2% were physicians. Age and working experience did not differ between professionals working in deprived and non-deprived neighbourhoods. Professionals working in a deprived neighbourhood had, on average, less client related activities per week and a lower number of consulted new-borns a year, than those working in a non-deprived neighbourhood.

**Table 1:** Respondents’ characteristics stratified by deprived and non-deprived neighbourhoods (*n* = 89)

Neighbourhood status	Deprived neighbourhood			Non-deprived neighbourhood		
	n	Percentage		n	Percentage	
Profession (% nurses)	11	61%		52	73%	
	n	Mean (SD)	Range	n	Mean (SD)	Range
Age (in years)	18	46.9 (10.1)	22–60	71	47.5 (0.5)	26–63
Working experience PCHC (in years)	18	16.2 (9.1)	1–28	71	16.3 (8.9)	1–38
Working experience in current position (in years)	18	13.8 (9.0)	1–28	71	14.4 (7.7)	1–35
Client-related activities (in number of days per week)	18	2.7 (0.8)	2–5	71	3.1 (0.7)	1–4
Consultations with new-borns (estimated number in the previous year)	16	120.9 (75.2)	50–350	68	132.7 (87.0)	40–450

Table 2 shows that 36% of the respondents encountered vulnerable families a couple of times a month and 30.3% encountered these families a couple of times a week, in the previous year. Professionals working in deprived neighbourhoods encountered vulnerable families significantly more often (*p* value = 0.025). Most of the respondents, 47.2%, experienced severe communication problems with clients several times a year, and 32.6% a couple of times a month. This percentage was significantly higher for professionals in deprived versus non-deprived neighbourhoods (*p* value = 0.001). With respect to the availability of guidelines or protocols for postnatal screening, 83.1% of the professionals indicated that these were available or being developed. Nine percent indicated that protocols were not available in their organisation and 2.2% did not know. This result did not differ between professionals working in deprived and non-deprived neighbourhoods (*p* value = 0.781).

**Table 2:** Current risk screening in PCHC according to PCHC physicians and nurses (*n* = 89)

	Never n (%)	Couple of times a year n (%)	Couple of times per month n (%)	Couple of times per week n (%)	Every day n (%)	Multiple times a day n (%)
Encountering families with non-medical risk factors, in the previous year	0 (0)	8 (9)	32 (36)	27 (30.3)	13 (14.6)	9 (10.1)
Severe communication problems with families during consultations, in the previous year	3 (3.4)	42 (47.2)	29 (32.6)	10 (11.2)	4 (4.5)	1 (1.1)

To assess current knowledge on risk factors influencing a child’s growth and development, respondents could indicate whether they thought a certain risk factor influences either growth or development of a young child. Table 3 shows the percentage of respondents who gave the correct answer, based on recent literature, which varied from 39.3 to 98.9%. Professionals working in non-deprived neighbourhoods had significantly better knowledge of financial problems and child overweight/obesity than those working in deprived neighbourhoods. However, for most questions no significant differences were found.

**Table 3:** Knowledge of PCHC professionals on risk factors for growth and developmental problems (*n* = 89)

Non-medical risk factor	Correct answer [reference]	Number of correct answers on the risk of overweight/ obesity				Correct answer [reference]	Number of correct answers on the risk of developmental problems			
		Deprived (*n* = 18)	Non-deprived (*n* = 71)	*p* value	*Total* *n (%)*		Deprived (*n* = 18)	Non-deprived (*n* = 71)	*p* value	*Total* *n (%)*
Parental smoking	Yes [37]	8	27	0.406	*35 (39.3)*	Yes [38, 39]	17	63	0.418	*80 (89.9)*
Parental drug use^a^	*NA* ^*a*^	*NA* ^*a*^	*NA* ^*a*^	*NA* ^*a*^	*NA* ^*a*^	Yes [26, 27]	18	69	0.635	*87 (97.8)*
Family income	Yes [40]	17	68	0.602	*85 (95.5)*	Yes [41]	17	59	0.205	*76 (85.4)*
Parental relationship problems^a^	*NA* ^*a*^	*NA* ^*a*^	*NA* ^*a*^	*NA* ^*a*^	*NA* ^*a*^	Yes [42]	18	70	0.789	*88 (98.9)*
Domestic violence	Yes [43]	14	43	0.138	*57 (64.0)*	Yes [42, 44]	18	70	0.798	*88 (98.9)*
Maternal overweight	Yes [29]	18	70	0.798	*88 (98.9)*	Yes [45]	12	43	0.424	*55 (61.8)*
Maternal alcohol abuse	No [46]	11	36	0.301	*47 (52.8)*	Yes [6]	18	65	0.246	*83 (93.3)*
Financial problems	Yes [47]	17	51	0.035	*68 (76.4)*	Yes [41]	17	63	0.418	*80 (89.9)*
Lack of social support	Yes [48]	18	59	0.054	*77 (86.5)*	Yes [49]	18	69	0.635	*87 (97.8)*

^a^NA: not applicable; in our literature search no articles were found which addressed this risk factor in association with growth problems

With regard to how many times in the previous year respondents performed screening on the prelisted non-medical risk factors, no significant difference was found in professionals working in deprived versus non-deprived neighbourhoods. Most of the respondents discussed smoking (68,2%), drug use (65,9%) and alcohol consumption (61,2%) in every consultation. Maternal weight was discussed the least by PCHC professionals (21.2% in none of the consultations). Domestic violence was not discussed often either; 11.2% of the professionals never discussed this topic during a consultation.

When encountering non-medical risk factors during a first consultation with a new-born baby, 12.2% did never offer an intervention, whereas 10% did always intervene. These interventions consisted of additional consultations by PCHC or referral to another healthcare professional. Most of the constraints for not offering an intervention were client related (82.4%) (e.g. financial restrictions or the prolonged traveling time to a care facility), less were related to healthcare professional restrictions (too little time during consultations) or to the intervention itself (such as waiting lists).

Healthcare professionals most often consulted by PCHC, in case of clients with non-medical risk factors, were social workers (15.3% in more than 50% of the clients) and Youth Welfare Service specialists (14.1% in more than 50% of the clients). Least consulted were gynaecologists and midwifes. No significant differences were found between professionals working in deprived and non-deprived neighbourhoods.

**Fig. 3:**
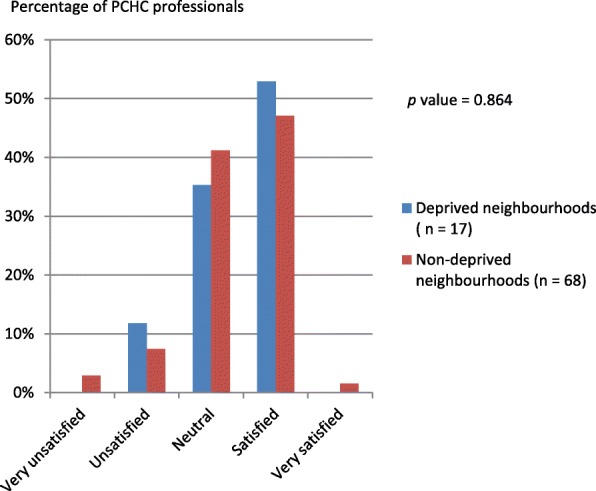
Opinion of PCHC professionals on current postnatal screening for non-medical risk factors (*n* = 85, missing = 4)

**Fig. 4:**
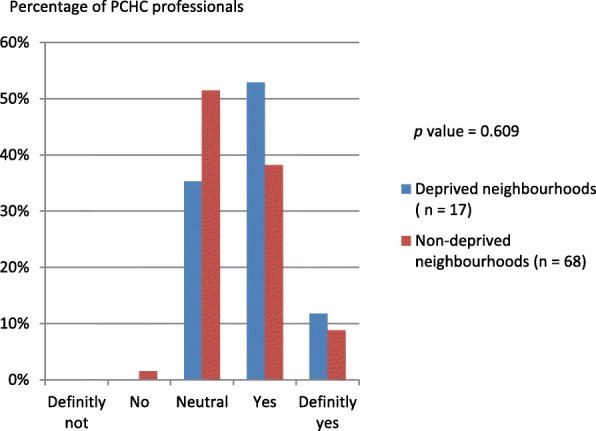
Opinion of PCHC professionals regarding the need for a structured postnatal risk assessment (*n* = 85, missing = 4)

Figure 3 represents the opinion of the respondents on current postnatal risk screening in their organisation, stratified by neighbourhood status. Most of the respondents (49.4%) were (very) satisfied and 40% of the respondents had no opinion. When it comes to the need for a structured postnatal risk assessment, most of the respondents were in favour (50.6%) of such an assessment and 48.2% had no opinion. This finding did not significantly differ between professionals working in deprived or and non-deprived neighbourhoods (Fig. 4), neither did it differ between physicians and nurses.

Regarding which healthcare professional should be the primary caregiver for families with non-medical risk factors (multiple answers were allowed), PCHC professionals favoured the general practitioner (62.2%), social work (75.3), PCHC physicians (78.8%), PCHC nurses (91.8%) and the district teams (89.9%), in which, in some municipalities, PCHC is a part of. In a district team, healthcare and social care professionals from a certain neighbourhood collaborate, in order to support clients close to home. In contrast, 82.4% of the respondents did not favour the gynaecologist or the paediatrician, and 74.1% did not favour the midwife as the primary caregiver. This opinion did not differ significantly between nurses and physicians or between professionals working in deprived and non-deprived neighbourhoods. (The full list of considered healthcare professionals can be found in Additional file 1: Table S1).

When it comes to the healthcare professional with whom the respondents would like to intensify collaboration, 67.1% mentioned district teams and 62.4% the general practitioner. In contrast, the majority did not feel the need to intensify collaboration with gynaecologists or the paediatrician (71.8 and 67.1%, respectively). This opinion did not significantly differ between PCHC nurses and PCHC physicians, and neither between neighbourhoods. (The full list of considered healthcare professionals can be found in Additional file 1: Table S2) 18.8% of the PCHC professionals did indicate they received handover from midwifes for every client and 25.9% received handover from maternity care for every client. For the other professions these percentages were lower. Details on smoking and substance abuse (drugs and alcohol) during pregnancy were most frequently missing in the handover, whereas almost all information can be registered in the PCHC client file.

## Discussion

The aim of this survey was to identify current Dutch PCHC risk screening practices and care for non-medical risk factors, during the postnatal period. Additionally, we studied different views and needs of professionals working in deprived and non-deprived neighbourhoods, the content of handover and their collaboration with other healthcare professionals.

Our study shows that PCHC professionals encounter clients with non-medical risk factors quite often, especially those working in deprived neighbourhoods. The importance of screening for non-medical risk factors seems to be recognised by PCHC professionals: most respondents often screen for important non-medical risk factors and they consider the care for vulnerable families as their responsibility. This corresponds with the development of Dutch PCHC guidelines and protocols, e.g. on parenting support, psychosocial problems, nutrition and eating habits and prevention of overweight. Although many PCHC professionals were satisfied with the current risk screening practices within their own organisation, half of the professionals feels the need for a structured postnatal risk assessment. This result did not significantly differ between professionals working in deprived or non-deprived neighbourhoods or between physicians and nurses. Neither did this need differ between professionals working in an organisation where a protocol was available or not. An explanation for this result may be, that most PCHC professionals are aware of non-medical risk factors and are satisfied with current practice, but that they screen without an official, national guideline or instrument. Johansen et al. [32] showed that a structured assessment of motor development in infants was well received by PCHC nurses, as they valued that working with this instrument increased the quality of care provided.

This study shows that PCHC professionals receive relatively few handovers from obstetric care professionals. Most handover is obtained from midwifes and maternity care, though not for every client. Moreover, essential information in the handover on prenatal and early postnatal smoking and substance abuse is often lacking, only one third of the professionals indicated that this information was ‘always available’ in handover documents.

Collaboration between healthcare professionals is advocated to improve patient outcomes [33] and enhances the quality of care given to individuals and groups in communities [34]. Poutianen et al. [35] showed that PCHC nurses’ understanding of the role of family characteristics could be valuable in promoting multidisciplinary work in healthcare. Collaboration between Dutch PCHC and other healthcare professionals exists but still is quite rare. District team members were involved most often, which may be due to the fact that in some municipalities, PCHC is part of the district teams.

### Limitations and strengths

A limitation of this study is that selection bias might have occurred, because of the non-random selection of participating PCHC organisations. Participating PCHC organisations might have been more eager to join the study because they were already more involved in postnatal non-medical risk screening. The response rate of the professionals within an organisation varied from 15.6% up to 100% between the PCHC organisations in this study. Nevertheless, we almost reached our target of completed questionnaires, having to rely on intermediates for the distribution of the questionnaire. The low response rate in some organisations could also be due to selection bias, as PCHC professionals who are more interested in the topic could be more willing to contribute to the survey. Another limitation of our study may be recall bias, which is a well-known restraint of survey studies. Our results show that most respondents can rely on protocols or local guidelines concerning risk screening. This might also have caused participants to respond with socially desirable answers due to their knowledge on certain risk factors, but not representing their current daily practice.

A strength of our study is that eight out of 36 PCHC organisations in the Netherlands participated. These eight organisations are likely to be a good reflection of PCHC organisations nationally, covering different areas in the south, north, east and west of the country and both rural and urban municipalities. Moreover, the mean age in our sample (47.0 years; SD 10.5) and the physician/nurse ratio (0.41, drawn from Table 1) are consistent with the results of Jambroes et al., who published an overview of the workforce of the Dutch PCHC services and who found a mean age of 48.0 (SD 10.2) years and a physician/nurse ratio of 0.48 [23]. This might indicate a good generalisability of our study, with a slight overrepresentation of nurses. However, since no significant differences between answers from physicians and nurses were found, this probably did not bias our results.

## Conclusion

This study shows that postnatal screening for non-medical risk factors is part of current practice of Dutch PCHC professionals, regardless the kind of neighbourhood they are deployed. They consider screening for non-medical risk factors as their responsibility. This study, emphasizes the need felt for a structured, evidence-based, postnatal risk assessment including non-medical risk factors, as well as the need for an intensified collaboration with other healthcare professionals. In a world where family engaged care [36] is integrated in health care policy and practice, strengthening collaboration between healthcare professionals is necessary. A structured postnatal risk assessment focussing on child characteristics, as well as parental and environmental characteristics contributes to this multilevel approach.

### Implications of this study

The management of PCHC organisations should invest in strengthening collaboration with other healthcare providers in a neighbourhood or municipality. Inter-professional collaboration across organisational boundaries is of utmost importance, especially for vulnerable families. Family engaged care and structured risk assessment for growth and developmental problems should become general practice in PCHC.

## Additional file

Additional file 1 **Table S1.** Respondents’ opinion on which healthcare professional should care for families with non-medical risks (*n* = 85). List of healthcare professionals considered, to care for families with non-medical risk factors and PCHC professional opinion on who should be responsible to care for these families. **Table S2.** Respondents’ opinion on with which healthcare professional collaboration should be intensified (*n* = 85). List of healthcare professionals considered, with whom PCHC professionals could collaborate with and their opinion with which healthcare professional collaboration should be intensified. (DOCX 14 kb)

## Abbreviations

NIVEL: Institute for health services research in the Netherlands
PCHC: Child preventive healthcare
SGA: Small for gestational age
