# Supplementary material for: Postnatal screening and care for non-medical risk factors by preventive child healthcare in deprived and non-deprived neighbourhoods
Source: BMC Health Serv Res. 2018 Jun 8;18:432. doi: 10.1186/s12913-018-3243-2 (PMC5994004; doi:10.1186/s12913-018-3243-2)
Supplement: Supplementary file 1 — Table S1. Respondents’ opinion on which healthcare professional should care for families with non-medical risks (n = 85). List of healthcare professionals considered, to care for families with non-medical risk factors and PCHC professional opinion on who should be responsible to care for these families. Table S2. Respondents’ opinion on with which healthcare professional collaboration should be intensified (n = 85). List of healthcare professionals considered, with whom PCHC professionals could collaborate with and their opinion with which healthcare professional collaboration should be intensified. (DOCX 14 kb) [file 12913_2018_3243_MOESM1_ESM.docx]

Additional file

**Table S1.** Respondents’ opinion on which healthcare professional should care for families with non-medical risks (n=85).

| **Health care professional** | **Yes, n (%)** | **No, n (%)** |
| --- | --- | --- |
| Midwife | 22 (25.9) | 63 (74.1) |
| Gynaecologist | 15 (17.6) | 70 (82.4) |
| General Practitioner | 52 (62.2) | 33 (38.8) |
| Paediatrician | 15 (17.6) | 70 (82.4) |
| Social work | 64 (75.3) | 21 (24.7) |
| Maternity care | 19 (22.4) | 66 (77.6) |
| PCHC Physician | 67 (78.8) | 18 (21.2) |
| PCHC Nurse | 78 (91.8) | 7 (8.2) |
| District Teams | 73 (85.9) | 12 (14.1) |
| Youth Social Services | 46 (54.1) | 39 (45.9) |
| Others: social team, youth team, medical social work, parenting support team, pre-primary education | 12 (14.1) | 73 (85.8) |

**Table S2.** Respondents’ opinion on with which healthcare professional collaboration should be intensified (n=85).

| **Healthcare professional** | **Yes, n (%)** | **No, n (%)** |
| --- | --- | --- |
| Midwife | 45 (52.9) | 40 (47.1) |
| Gynaecologist | 24 (28.2) | 61 (71.8) |
| General Practitioner | 53 (62.4) | 32 (37.6) |
| Paediatrician | 28 (32.9) | 57 (67.1) |
| Social work | 43 (50.6) | 42 (49.4) |
| Postnatal care | 35 (41.2) | 50 (58.8) |
| District Teams | 57 (67.1) | 28 (32.9) |
| Youth Welfare Services | 42 (49.4) | 43 (50.6) |
